# Systemic Air Embolism Following Computed Tomography-Guided Lung Biopsy

**DOI:** 10.7759/cureus.5408

**Published:** 2019-08-17

**Authors:** Haisam Abid, Amrat Kumar, Nadir Siddiqui, Bruce Kramer

**Affiliations:** 1 Internal Medicine, Bassett Medical Center, Cooperstown, USA; 2 Internal Medicne, Bassett Medical Center, Cooperstown, USA; 3 Critical Care, Bassett Medical Center, Cooperstown, USA

**Keywords:** air embolism, lung nodule, ct guided lung biopsy

## Abstract

A 61-year-old male with a history of poorly differentiated squamous cell carcinoma of tongue who completed chemo-radiation was found to have bilateral lung nodules on follow-up positron emission tomography (PET) scan. He underwent computed tomography (CT)-guided lung biopsy. Sequential chest scans done during the procedure showed air-fluid level in the left ventricle, suggestive of air embolism. He was hemodynamically stable during the procedure, however at the end of the procedure he developed right-sided face and arm weakness with aphasia. Emergent CT scans including angiography of head and neck were done which did not show any bleed and was also negative for any air in intracranial vasculature. Patient was treated with 100% oxygen. His neurological symptoms resolved in 30 minutes and he was subsequently admitted to intensive care unit (ICU) for further management. Six hours later, repeat CT of chest was done which showed resolution of air embolism.

## Introduction

Air embolism is an uncommon but potentially catastrophic complication that occurs because of air entry into vessels which can be either arterial or venous. Most common causes include surgery, trauma, vascular interventions, and barotrauma from mechanical ventilation and diving. Here, we present a rare case in which patient developed air embolism after computed tomography (CT)-guided lung biopsy.

## Case presentation

**Figure 1:**
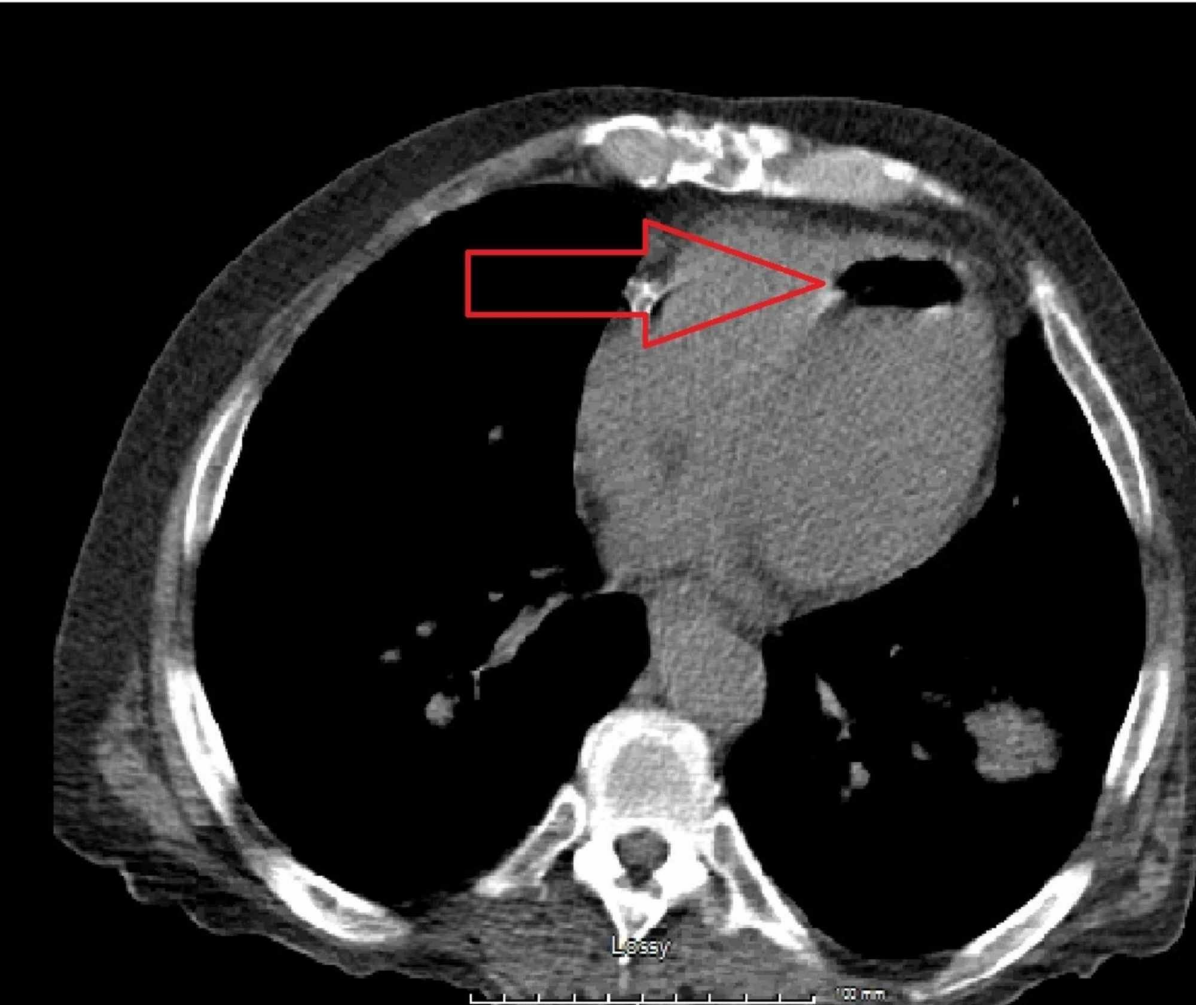
Computed tomography (CT) scan of chest showing air embolism in left ventricle

A 61-year-old male with a medical history of poorly differentiated squamous cell carcinoma of tongue treated with chemo-radiation was found to have two bilateral lung nodules, largest nodule measuring 1.1 x 1.5 cm in size, on follow-up positron emission tomography (PET) scan. He presented to radiology department for CT guided lung biopsy. An 18-gauge coaxial Tru-Cut biopsy needle was placed in the left lower lung nodule. Needle placement was assessed with additional CT images. Three passes were made and samples were sent for analysis. Sequential chest scans done during the procedure showed air-fluid level in the left ventricle, suggestive of air embolism (figure 1).

**Figure 2:**
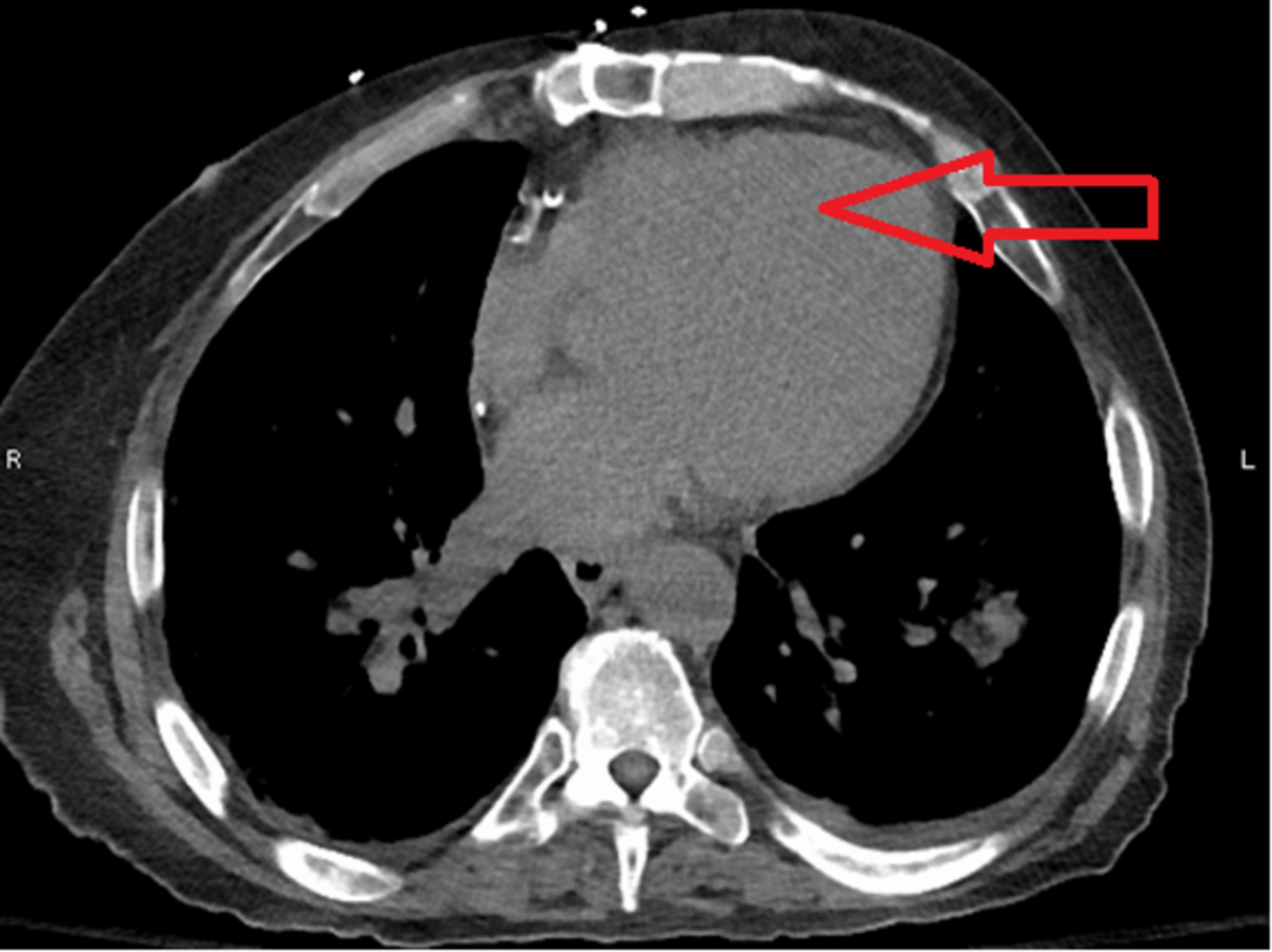
Computed tomography (CT) scan of chest showed resolution of air embolism in left ventricle

The patient developed right-sided face and arm weakness with aphasia. CT head without contrast, CT angiography of head and neck were done. CT head did not show any bleed and CTA head and neck was negative for any air in intracranial vasculature. His neurological symptoms are most likely related to air embolism going into his nervous system. He was placed in the Trendelenburg position and was given 100% oxygen via high flow nasal cannula. His neurological symptoms resolved in 30 minutes and he was subsequently admitted to the intensive care unit (ICU). Repeat CT of chest was done which showed resolution of air embolism (figure 2).

## Discussion

Due to widespread use of imaging, asymptomatic lung nodules are very common incidental finding [1]. Despite all the advancement in imaging studies, these nodules are often classified as indeterminate and tissue diagnosis is usually required. CT guided percutaneous transthoracic biopsy of the lung is a well-known procedure for definitive diagnosis of lung nodules [2].

The most common complication of percutaneous lung biopsies is pneumothorax [3]. Risk factors for pneumothorax include severe underlying chronic obstructive lung disease, longer needle path > four centimeters, sub-pleural and smaller lesions. The reported incidence of air embolism after CT guided transthoracic lung biopsy ranges from 0.02% to 0.07%. The actual incidence may be higher because it is often asymptomatic and not reported [2-4]. Air embolism typically causes clinical symptoms due to ischemic effects of gas within coronary and cerebral circulations and include neurological symptoms such as focal neurologic deficits, seizures, and altered levels of consciousness and manifestations related to heart ischemia such as chest pain, hypotension, dyspnea, and cardiopulmonary arrest in extreme cases [2].

There are different mechanisms by which air can enter into the systemic circulation and can cause significant morbidity or mortality. Air can be introduced into the systemic circulation due to communication between the pulmonary vein and the atmosphere. Sometimes, air can enter into the systemic circulation if there is a communication between air containing spaces and pulmonary veins due to broncho-venous fistula. Third mechanism is if air traverses the pulmonary microvasculature and enters into the pulmonary venous circulation [5-6].

Early recognition of systemic air embolism is important because simple treatment measures may lead to complete resolution. Usually, it is asymptomatic, but small amount of air such as 2 mL in the cerebral circulation can be dangerous, and 0.5 to 1.0 mL of air entry into the pulmonary venous system can lead to coronary embolism and cardiac arrest [6-7]. 

The treatment of suspected or confirmed arterial air embolism includes immediately stopping the procedure, give 100% oxygen supplementation and patient placed in right lateral decubitus position to prevent air from entering the systemic circulation by keeping the left ventricular outflow tract in a nondependent position away from the aorta; it will trap air in the left ventricle and prevent air to go into the brain or other important vasculature. After initial supportive therapy, if possible, the patient should be placed in hyperbaric oxygen chamber as it has been shown effective treatment option to cause the exchange of oxygen for nitrogen in the air bubbles [6].

## Conclusions

Air embolism should be a differential diagnosis in patients with neurological symptoms especially after a surgical procedure, central line placement, or removal. Early identification and treatment are important in air embolism. Physicians should be aware of the clinical situations which can lead to air embolism and all precautionary measures should be taken, as prevention is the best treatment.
